# Breathing New Life to Ancient Crops: Promoting the Ancient Philippine Grain “Kabog Millet” as an Alternative to Rice

**DOI:** 10.3390/foods9121727

**Published:** 2020-11-24

**Authors:** Joan Oñate Narciso, Laura Nyström

**Affiliations:** Laboratory of Food Biochemistry, Department of Health Sciences and Technology, Institute of Food, Nutrition and Health, ETH Zürich, Schmelzbergstrasse 9, CH-8092 Zurich, Switzerland; joan.narciso@hest.ethz.ch

**Keywords:** *Panicum miliaceum*, ancient grains, nutritional quality, dietary fibre, protein, essential amino acids, phenolic acids, carotenoids, antioxidant properties, rice

## Abstract

Consumption of underutilised ancient crops has huge benefits for our society. It improves food security by diversifying our staple foods and makes our agriculture more adaptable to climate change. The Philippines has a rich biodiversity and many plant species used as staple foods are native to the Philippines. An example of ancient Philippine crops is the kabog millet, an ecotype of *Panicum miliaceum.* There is a dearth of information about its uses and properties; hence, in this study, the nutritional quality of kabog millet was evaluated. The total starch, % amylose, ash, dietary fibre, proteins, essential amino acid profile, phenolic acids, carotenoids, tocopherols, and the antioxidant properties of its total phenolic acid extracts were compared to four types of rice (white, brown, red, and black) and a reference millet, purchased from local Swiss supermarkets. Our analyses showed that kabog millet has higher total dietary fibre, total protein, total phenolic acids, tocopherols, and carotenoids content than white rice. It also performed well in antioxidant assays. Our results indicate that kabog millet is a good alternative to rice. It is hoped that the results of this study will encourage consumers and farmers to diversify their food palette and address food insecurity.

## 1. Introduction

Cultivating ancient crops has huge benefits for our society. It improves food security by diversifying our staple foods, provides a greater range of options to address climate change, optimises land resources by cultivating soils unsuitable for the world’s major crops, promotes access to better nutrition for communities, especially in the developing regions of the world, and generates income opportunities for small- and medium-scale farmers [1]. The Philippines has a rich biodiversity and cultural heritage from its indigenous peoples. Many plant species used as staple foods, such as adlay (a type of grain), balayang (a wild banana), and landing (native tapioca) are native to the Philippines. Unfortunately, the knowledge about their uses and cultivation is slowly being forgotten with the demise of indigenous cultures. An example of ancient Philippine crops is the kabog millet.

**Figure 1:**
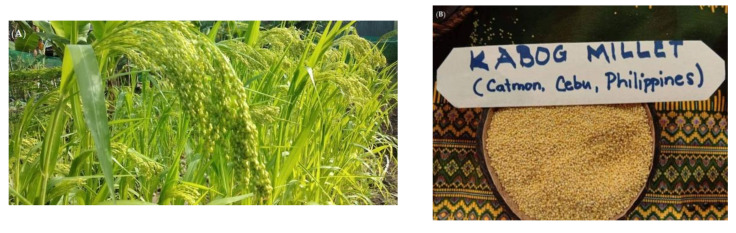
Kabog millet plants (**A**) and kabog millet grains (**B**). Photo credits: Mrs. Rosaflor Montecillo Estenzo for the kabog millet plants and Cebu Farmers Market for the kabog millet grains.

Little is known about the kabog millet (Figure 1), considered an ecotype of *Panicum miliaceum* L., and the only known proso millet existing in the Philippines. Morphological studies on two kabog millet cultivars grown in Cebu, Philippines, placed them under the *Panicum patentissium* and *Panicum compactum* subspecies [2], but genetic studies to confirm this classification remain missing. Kabog millet is sometimes mistakenly referred to as “dawa”, meaning “bird seeds,” but “dawa” is rather a term used for foxtail millet (genus *Setaria*), for which the grains come in darker and lighter brown colours. Kabog millet once grew wild and in abundance in the mountains of Catmon, Cebu, one of the central Visayas Islands of the Philippines, from locally acquired seeds. Before the Spanish colonisers came to the Philippines in the 16th century, kabog was the native cereal staple food of the Cebuanos. This millet is used to make “budbud kabog”, a term for a yellow-coloured tube-cake [3]. It gets its name from a local folklore: a farmer once discovered millet grains scattered on a cave floor. The cave bats (kabog, in the local language) used the millet as food. Thinking that the seeds are edible, he cooked them but found his recipe unpalatable. He then experimented by pounding the millet seeds before cooking, and added sugar, creating a delicious dish, which became “budbud kabog” [3].

Since there is a lack of nutritional studies for kabog millet, it is unknown how it will compare with rice; however, other millet varieties were found to have high nutritive value and comparable to wheat and rice [4,5]. Millet proteins are also good sources of essential amino acids except lysine and threonine but are relatively high in methionine. In addition, millets are rich sources of phytochemicals and micronutrients [6,7].

Kabog is not usually sold in its raw form on the market, and in the only rare cases, as small quantities. A kabog millet farmer can harvest around one cavan (a Philippine unit of measurement for dry capacity, which weighs approximately 50 kg) each harvesting season (Estenzo, personal communication). There are at least 50 kabog millet farmers in Cebu, Philippines. This translates to at least 2.5 metric tonnes of kabog millet harvested each season. Overall, the production of the kabog is dwindling. It is not promoted as a profitable crop, and the government encourages cultivation of higher yielding varieties. Traditional, historical crops like kabog are disregarded and are often overlooked. Hence, a nutritional content analysis of kabog millet is needed to encourage farmers, consumers, and the government to consider it as an alternative to rice. Since rice has nutritional deficiencies, consumption of other types of cereals is highly recommended. We report here the nutritional quality of kabog millet compared to different rice types (white, brown, red, and black), and a reference millet. Because millets are not commonly cultivated in the Philippines, we used the reference millet that can be bought from local supermarkets in Switzerland for comparison with the kabog millet. This study aims to promote kabog millet consumption in the Philippines and potentially, in other rice-consuming countries in the world, so that it can be saved from near extinction.

## 2. Materials and Methods

### 2.1. Samples

White rice (Thai Hom Mali Rice, Thailand), brown rice (Healthy Grain, Thai Hom Mali Brown Rice “Jasmine Brown Rice”, Thailand), red rice (Healthy Grain, Red Cargo Rice, Thailand), and black rice (Healthy Grain, Black Cargo Rice “Riceberry”, Thailand) were purchased from New Asia Market Zürich. The reference millet, which was grown in Austria, was purchased from Migros, a Swiss supermarket (Zürich, Switzerland). Unmilled (whole grain) kabog millet and milled (dehulled) kabog millet were purchased from two farmers in Cebu, Philippines: (1) Mrs. Rosaflor Montecillo Estenzo, Multiple farmers/stock from cooperative selling, Location: Purok Tambis, Baranggay Agsuwao, Catmon, Cebu, Date harvested: 2nd week of August 2019; (2) Mr. Nolito Ares, Farmers association, Location: Baranggay Agsuwao, Catmon Cebu, Date harvested: 1st week of August 2019.

### 2.2. Grinding

The four types of rice grains, the reference millet, and the whole and dehulled kabog millet samples were air-dried at ambient temperature for three days in aluminium dishes (diameter: 6.0 cm; height/depth: 2.5 cm). After three days, the samples were ground using a Retsch ultracentrifugal mill ZM200 (speed: 12,000 rpm, particle size: 500 µm) (Retsch GmbH, Haan, Germany), vacuum-sealed, and frozen until further analysis.

### 2.3. Nutritional Content Analysis

For the succeeding nutritional content determination, the analyses were performed on a dry matter basis.

#### 2.3.1. Starch, % Amylose, Dietary Fibre

Total starch (K-TSTA-100A), amylose/amylopectin assay (K-AMYL), and dietary fibre (K-TDFR-200A) kits were purchased from Megazyme Ltd. (Bray, Ireland), and the analyses were performed according to the manufacturer’s protocols.

#### 2.3.2. Ash

Ash content analysis was performed according to [8], with slight modifications: Flour samples and porcelain crucibles were dried in the oven at 130 °C for 90 min, then stored in the desiccator. The weight of each crucible was determined. For each flour type, 1.5 g was weighed in a porcelain crucible and the weight of the crucible with the flour was recorded. The samples were placed in a muffle furnace at 600 °C for 5 h. The residues were weighed after cooling. The % Ash was calculated using the formula:

% Ash = (M_ash_/M_sample_) × 100

where M_ash_ is the weight of the residue after ashing in the muffle furnace and M_sample_ is the weight of the dry flour sample before ashing.

#### 2.3.3. Total Protein, Amino Acid Profiling

The ground samples were sent to AgroVet-Strickhof in Lindau, Switzerland, for Dumas analysis of total nitrogen (N) content. To obtain the total protein content, the %N were multiplied with 5.95 (rice samples) and 5.83 (millet samples) [9]. Prior to amino acid profiling, total proteins were extracted using the protocol of [10] with slight modifications: 50 mg of ground sample was weighed into 1.5-mL microfuge tube, and 1 mL of borate extraction buffer (12.5 mM sodium borate, pH 10, 1% SDS, and 2% 2-mercaptoethanol) was added to each tube, and the tubes were vortexed. The tubes were incubated at 37 °C overnight with shaking. After incubation, the samples were centrifuged at room temperature for 15 min. The supernatants were collected and sent to the Functional Genomics Center Zürich (Zürich, Switzerland) for amino acid profiling. In total, 16 amino acids were tested. Sample analysis was performed on a Waters ACQUITY ultra-performance liquid chromatography (UPLC) system (Waters, Baden-Dättwil, Switzerland) and the derivatised amino acids were detected on a Waters QDa single quadrupole mass detector (Waters, Baden-Dättwil, Switzerland) in positive mode.

#### 2.3.4. Carotenoids

The method in [11] was followed for carotenoids analysis. Due to light sensitivity of carotenoids, the samples and standards were protected from light by using 50-mL dark Falcon tube into which 500 mg of ground sample was weighed. To each tube was added 50 µL of internal standard solution [0.07 mg/mL: β-apo-8′-carotenal (purchased from Sigma-Aldrich, St. Louis, MI, USA) in methyl *tert-*butyl ether (MTBE)/methanol, 2:8 *v*/*v*] using a Hamilton syringe. After, 5 mL of 1-butanol was added to each tube with a glass pipette. The samples were vortexed quickly and were put in a sonicator for 15 min. They were stirred for 30 min on a magnetic stirrer and were sonicated again for 15 min. The samples were stirred for another 30 min, after which they were centrifuged at room temperature for 3 min at 4000 rpm. The supernatant (1 to 1.5 mL) was filtered through a hydrophilic syringe filter [13 mm, polytetrafluoroethylene (PTFE), 0.45 µM] (BGB, Boeckten, Switzerland) into an amber-coloured high-performance liquid chromatography (HPLC) vial. The samples were stored at −20 °C and light protected until further analysis with HPLC.

The HPLC parameters were as follows: Flow rate was 1.0 mL/min, injection volume was 50 μL, temperature of column was at 35 °C, diode-array detection (DAD) was at 450 nm, and the separation was within 18 min. The samples were run using the HPLC column YMC Carotenoid, S-3µm, 100 × 4.6 mm with the pre-column YMC Carotenoid, S-3µm, 10 × 4.0 mm (YMC Schweiz GmbH, Basel, Switzerland) in reverse phase high-performance liquid chromatography (RP-HPLC) Agilent 1200 (Agilent Technologies, Basel, Switzerland). The composition of the eluent and the gradient program were A: Methanol/MTBE/Milli-Q water, (81:15:4); B: MTBE/methanol (90:10): with a gradient from 100% to 10% A (0–15 min), and from 10% to 100% A (15–18 min).

#### 2.3.5. Tocopherols

The tocopherols analysis was done following the method in [12]. For each milled sample, 0.5 g was weighed into a 30-mL pyrex glass tube with a teflon screw cap. To each tube was added 0.1 g ascorbic acid as an antioxidant. Samples were analysed in groups of four: three times with internal standard, one time without. The samples were suspended in 5.0 mL ethanol and 2.0 mL water. The internal standard, 50 μL δ-tocopherol (conc. 0.1 mg/mL, purchased from Sigma-Aldrich), was added using a Hamilton syringe, and mixed with a vortex mixer. The tubes were let to stand for 10 min and were then flushed with nitrogen for 20 s to remove the oxygen. Half mL (0.5 mL) of 50% potassium hydroxide (KOH) solution (*w/v*, 50 g KOH in 100 mL MilliQ water) was added to each tube with a magnetic stirrer. The samples were mixed thoroughly by vortexing. They were then put into a boiling water bath (100 °C) and allowed to saponify for 25 min under constant stirring. The tubes were cooled in a cold-water bath for around 10 min, after which 2.5 mL water and 2.5 mL ethanol were added. Tocols were extracted by adding 10 mL *n*-hexane:ethylacetate (8:2, *v*/*v*). The tubes were placed in a tumbler mixer to shake for 10 min. After shaking, the tubes were let to stand until the organic and non-organic phases separated. The upper organic layer was quantitatively transferred into a 100-mL separation funnel using a pasteur pipette. The extraction was repeated twice. The organic extract in the separation funnel was washed with 1 mL of 10% sodium chloride (NaCl) solution. The lower layer was removed from each washing. The organic extract was transferred into a 100-mL round-bottom flask, and the solvent was evaporated using the rotary evaporator, with the water bath at around 60 °C and the pressure around 300 mbar. The lipid residue was resolubilised in *n*-hexane and quantitatively transferred into a 5-mL volumetric flask. The volumetric flask was filled with *n*-hexane. An aliquot of the sample (around 1.5 mL) was filtered into an HPLC vial using a 45-μm PTFE syringe filter. The vials were stored at −20 °C until HPLC analysis. For normal phase HPLC Agilent 1200 (Agilent Technologies, Basel, Switzerland), the following parameters were used: eluent *n*-hexane: 1,4-dioxane, 97:3 (*v*/*v*), flow rate 2 mL/min, injection volume 50 μL, temperature of column 30 °C, temperature of autosampler tray 4 °C, fluorescence detection (FLD) with excitation wavelength λ_ex_ 292 nm and emission wavelength λ_em_ 325 nm, isocratic separation within 25 min.

#### 2.3.6. Phenolic Acids

The method in [13] was adapted for the phenolic acid analysis. Total phenolic acids were extracted from 500 mg of ground flour, weighed in 50-mL Falcon tubes. One mL (1 mL) of internal standard [0.25 mg/mL 3,5-dichloro-4-hydroxybenzoic acid (from Sigma-Aldrich) in methanol] was added to each tube to spike the sample with 250 µg of standard, after which 20 mL of 2 M sodium hydroxide was added to release esterified and bound phenolic acids for 1 h at room temperature while constantly stirring on stir plate. To neutralise the samples, 4.0 mL concentrated hydrochloric acid and 10 mL ethyl acetate were added to each tube and were vortexed. After centrifuging the sample for 5 min at 4000 rpm, the supernatant was transferred into a fresh 50-mL Falcon tube using a Pasteur pipette. Extraction with hydrochloric acid and ethyl acetate was repeated and the supernatants were combined. The solvent was evaporated with a gentle nitrogen stream at 60 °C. The dried samples were dissolved in 1000 µL of HPLC eluent (methanol:water:formic acid, 90:8:2) and sonicated. The redissolved samples were transferred into HPLC vials using a syringe with a hydrophilic filter (see Section 2.3.4).

The RP-UHPLC parameters were as follows: flow rate 0.4 mL/min, injection volume 1 μL, column and autosampler at ambient temperature, DAD detection at 254, 280, and 325 nm. The samples were run using the ultra-high-performance liquid chromatography column (UHPLC-Column) ACQUITY UPLC^®^ BEH C18 (2.1 × 100 mm, particle size 1.7 µm) (Waters, Baden-Dättwil, Switzerland) in UHPLC 1290 Infinity II Series (Agilent Technologies, Basel, Switzerland). The composition of the eluent and the gradient program were A: Methanol:formic acid (90:2); B: Milli-Q water:formic acid (88:2): from 0% to 20% A (0–5 min), at 20% A (5–6 min), from 20% to 50% A (6–12.3 min), at 50% A (12.3–13.2 min), from 50% to 100% A (13.2–15.3 min), at 100% A (15.3–16.9 min), from 100% to 0% A (16.9–19.1 min). 

#### 2.3.7. Antioxidant Assays

For the antioxidant assays, the total phenolic acid extracts (Section 2.3.6) were tested. The 2,2-diphenyl-1-picrylhydrazyl (DPPH) radical scavenging activity method used in this study was modified from [14]. The DPPH solution was freshly prepared in methanol and was brought to a concentration of 112 μM in the reaction system. Pyrogallol in methanol (final concentration 66 μM) was used as the positive control. After quickly mixing the antioxidant (total phenolic acid extracts or pyrogallol) and DPPH, the absorbance at 517 nm was recorded immediately at 0 min and after 5 min using a Cary 100 spectrophotometre (Agilent Technologies, Basel, Switzerland). The %Radical scavenging activity (%RSA) was calculated as follows:

%RSA = [(A_0_ − A_t_)/(A_0_ − A_p_)] × 100

where A_0_ = absorbance (DPPH + sample or pyrogallol) at 0 min, A_t_ = absorbance (DPPH + sample) at 5 min and A_p_ = absorbance (DPPH + pyrogallol) at 5 min.

The 2,2’-azino-bis(3-ethylbenzothiazoline-6-sulfonic acid)(ABTS) Antioxidant Capacity Assay (KF01002) and the Oxygen Radical Absorbance Capacity (ORAC) Assay (KF01004) kits were from Bioquochem (Asturias, Spain), and the ABTS and ORAC antioxidant assays were performed using the total phenolic acid extracts following the manufacturer’s protocols. Calculations for %inhibition, Trolox equivalence antioxidant capacity (TEAC), and vitamin C equivalence antioxidant capacity (CEAC) were included in the manufacturer’s protocols.

#### 2.3.8. Fatty Acid Methyl Esters (FAME)

The fatty acid characterisation was performed only on whole kabog millet samples. To obtain the fatty acids from whole kabog millet, 5.0 g of ground flour was extracted with 20 mL *n*-hexane. The samples were vortexed for 2 min, sonicated for 10 min, then centrifuged at 4000× *g* at room temperature for 5 min. The supernatant was transferred to a new 50-mL Falcon tube, and the hexane was gently evaporated completely with nitrogen stream at room temperature. The residue was resuspended in 500 µL of *n*-hexane. To each replicate, 125 µL of the resuspended sample was added to 500 µL *n*-hexane and 300 µL 2M KOH in methanol, and the mixture was vortexed for 2 min. The samples were let to stand until the layers separate, and the upper layer was pipetted off and transferred to a gas chromatography (GC) vial for further gas chromatography-flame ionisation detector (GC-FID) and gas chromatography-mass spectrometry (GC-MS) analyses using the ThermoFisher Trace 1310 Series GC (ThermoFisher Scientific, Reinach, Switzerland). The GC standard used was Supelco 37 Component FAME Mix (Supelco).

### 2.4. Statistical Analysis

All experiments were carried out in triplicates unless otherwise stated and data were reported as mean ± standard deviation. The data in Figures were evaluated using a one-way ANOVA (*p* < 0.05) (https://astatsa.com/OneWay_Anova_with_TukeyHSD/). Differences among means in Tables were evaluated by Tukey’s HSD tests (*p* < 0.05) using the free statistical software R project, v. 3.6.3 (downloaded from https://cran.r-project.org/mirrors.html).

## 3. Results

The nutritional content and quality of kabog millet, both whole and dehulled, from two sources (see Section 2.1) were determined in comparison with rice (white, brown, red, and black), and a reference millet from a local Swiss supermarket. The parameters measured were total starch, % amylose, total dietary fibre, ash, total protein, essential amino acid profiles, phenolic acids, carotenoids, tocopherols, fatty acids, and DPPH, ABTS, and ORAC antioxidant assay activities.

### 3.1. Starch

The whole kabog millet sample 1 (51.6 ± 1.8 g/100 g sample) had comparable total starch with whole kabog millet sample 2 (47.4 ± 2.0 g/100 g sample). The same trend was noted in the dehulled kabog millet samples (Table 1). The total starch values of the whole and dehulled kabog millet samples were significantly lower than the rice samples and the reference millet. Among the rice samples, the white and brown rice samples had the highest total starch content, followed by the red and black rice samples.

For the % amylose (Table 1), the whole kabog millet sample 1 (16.4 ± 1.1%) had comparable value with whole kabog millet sample 2 (18.1 ± 1.0%) while the dehulled kabog millet sample 1 (19.1 ± 1.8%) had significantly higher % amylose than the dehulled kabog millet sample 2 (10.7 ± 1.3%). In general, the % amylose of the whole kabog and dehulled kabog samples except for dehulled kabog millet sample 2 were not significantly different to all rice samples and the reference millet (Table 1).

### 3.2. Dietary Fibre and Ash

For the dietary fibre, the whole kabog millet sample 1 (14.6 ± 0.6%) had comparable value with whole kabog millet sample 2 (13.8 ± 0.9%). Likewise, the dehulled kabog millet samples had similar values (sample 1 with 3.6 ± 0.6%; sample 2 with 4.0 ± 1.1%) as shown in Table 1. The whole kabog millet samples had significantly higher dietary fibre content than the dehulled kabog millet samples, all rice samples, and the reference millet.

The ash content of the whole kabog millet sample 1 (4.2 ± 0.0%) was significantly higher than the whole kabog millet sample 2 (4.1 ± 0.1%). On the other hand, the ash content of dehulled kabog millet sample 1 (1.0 ± 0.0%) was significantly lower than the dehulled kabog millet sample 2 (1.1 ± 0.0%). Overall, the whole kabog millet samples had significantly higher ash content than the dehulled kabog millet samples, all the rice samples, and the reference millet (Table 1).

### 3.3. Protein and Essential Amino Acids

In terms of total protein, the whole kabog millet sample 1 (12.0 ± 0.0 g/100 g sample) had comparable value as the whole kabog millet sample 2 (12.1 ± 0.0 g/100 g sample), while the dehulled kabog millet sample 1 (12.5 ± 0.0 g/100 g sample) had significantly lower content than the dehulled kabog millet sample 2 (12.9 ± 0.1 g/100 g sample) (Table 1). Overall, the total protein content of the whole kabog millet and the dehulled kabog millet samples were significantly higher than that of the rice samples and the reference millet.

**Figure 2:**
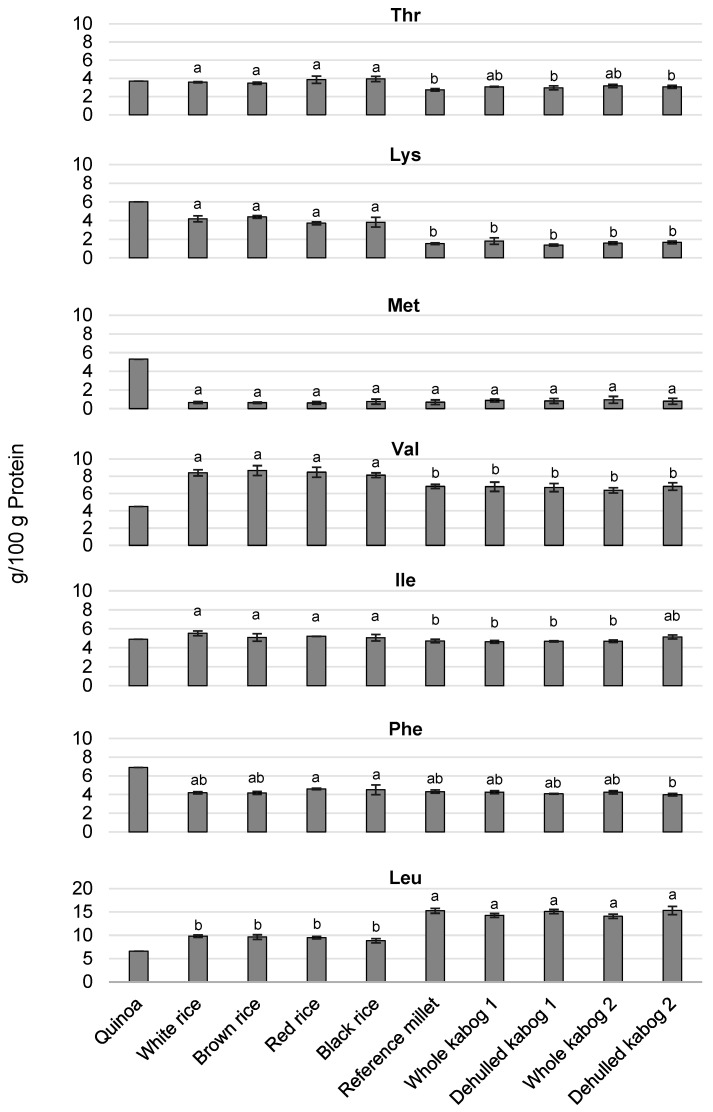
Essential amino acid content (g/100 g protein) of rice (white, brown, red, and black), reference millet, and kabog millet (whole and dehulled) samples compared to quinoa [15]. Kabog millet 1 refers to kabog millet obtained from farmer source 1, and kabog millet 2 refers to kabog millet obtained from farmer source 2. The values followed by the same letters are not significantly different at *p* < 0.05 using one-way ANOVA Tukey’s HSD.

The essential amino acids (excluding Trp) in the rice and millet samples were compared with quinoa, a pseudocereal known for its protein quality [15]. In terms of Thr, the whole kabog millet sample 1 (3.07 ± 0.04 g/100 g protein) had comparable value with the whole kabog millet sample 2 (3.16 ± 0.17 g/100 g protein) (*p* < 0.05). Likewise, the Thr content of the dehulled kabog millet sample 1 (2.96 ± 0.21 g/100 g protein) was not significantly different with the dehulled kabog millet sample 2 (3.06 ± 0.16 g/100 g protein) (*p* < 0.05) (Figure 2). In general, the dehulled kabog millet samples and the reference millet had significantly lower Thr content than the rice samples (*p* < 0.05). The Lys, Ile, and Val content were comparable between whole kabog millet samples, the dehulled samples, and the reference millet, but significantly lower than in the rice samples (*p* < 0.05). The Leu content was comparable between whole kabog millet samples, the dehulled samples, and the reference millet, but significantly higher than in the rice samples (*p* < 0.05). The Phe content was not significantly different between whole kabog millet samples, the dehulled samples, and the reference millet. However, the Phe content of the dehulled kabog millet sample 2 (3.99 ± 0.13 g/100 g protein) was significantly lower than that of red rice (4.61 ± 0.07 g/100 g protein) and black rice (4.51 ± 0.53 g/100 g protein) (*p* < 0.05). In terms of Met, there was no significant difference among all the samples. Quinoa, however, still has the highest content of Lys, Met, and Phe (Figure 2).

### 3.4. Carotenoids

The total carotenoid content of the whole kabog millet sample 1 (16.8 ± 0.4 µg/g) was not significantly different with the whole kabog millet sample 2 (15.7 ± 0.6 µg/g). The same comparable values were noted between dehulled kabog millet sample 1 (19.4 ± 0.3 µg/g) and dehulled kabog millet sample 2 (19.3 ± 0.5 µg/g). The whole kabog millet samples and the dehulled kabog millet samples had significantly higher total carotenoid content than the rice samples and the reference millet with the dehulled kabog millet samples having significantly higher total carotenoids than the rest of the samples (Table 2).

In terms of individual carotenoids, the whole kabog millet samples (11.6 ± 0.3 µg/g and 12.9 ± 0.3 µg/g) had significantly lower amounts of lutein than the dehulled kabog millet samples (14.5 ± 0.2 µg/g and 15.1 ± 0.2 µg/g) (*p* < 0.05). The reference millet had significantly lower lutein content (4.7 ± 0.2 µg/g) than the kabog millet samples (*p* < 0.05). The rice samples contained lower amounts of lutein than the kabog millet samples. Zeaxanthin was present in the reference millet (1.7 ± 0.1 µg/g), in both whole kabog millet samples 1 and 2 (3.4 ± 0.2 µg/g and 3.1 ± 0.1 µg/g, respectively), and in both dehulled kabog millet samples 1 and 2 (3.8 ± 0.4 µg/g and 4.0 ± 0.2 µg/g, respectively). The reference millet had lower lutein and zeaxanthin content than the kabog millet samples. In this study, zeaxanthin was not detected in the white, brown, and red rice samples. Black rice had zeaxanthin and β-cryptoxanthin, but the amount was low (≈0.1 µg/g for both carotenoids). The kabog millet samples also contained β-carotene, which were lower than black rice (1.4 ± 0.0 µg/g sample) (*p* < 0.05).

### 3.5. Tocopherols

The total tocopherol content (Table 2) of the whole kabog millet sample 1 (93.1 ± 9.0 µg/g) was not significantly different with that of the whole kabog millet sample 2 (99.4 ± 9.4 µg/g). Comparable total tocopherol contents were also noted between the dehulled kabog millet sample 1 (45.0 ± 5.3 µg/g) and the dehulled kabog millet sample 2 (50.4 ± 6.3 µg/g). The whole kabog millet samples had significantly higher total tocopherol content than the dehulled kabog millet samples, the reference millet, and the rice samples, except for black rice (120.0 ± 15.7 µg/g).

Among the tocopherols, γ-tocopherol and δ-tocopherol were present in significant amounts in the kabog millet and reference millet samples (*p* < 0.05). The γ-tocopherol values ranged from 29.8 ± 1.4 µg/g for the reference millet to 83.9 ± 7.2 µg/g for the whole kabog 2. On the other hand, the δ-tocopherol contents ranged from 5.6 ± 0.2 µg/g for the dehulled kabog 2 to 13.9 ± 2.3 µg/g for the whole kabog 2. The reference millet contained 7.4 ± 0.7 µg/g δ-tocopherol. The δ-tocopherol form was not detected in rice. However, γ-tocotrienol was the most abundant form in rice, with values ranging from 10.8 ± 0.1 µg/g (white rice) to 80.1 ± 9.8 µg/g (black rice). The tocopherol, γ-tocotrienol, was not detected in the whole and dehulled kabog millet and the reference millet.

### 3.6. Phenolic Acids

In terms of total phenolic acids (Table 2), the whole kabog millet sample 1 (1519.0 ± 47.1 µg/g) had significantly lower content than the whole kabog millet sample 2 (1606.6 ± 16.2 µg/g). On the other hand, comparable total phenolic acids were recorded between dehulled kabog millet sample 1 (264.7 ± 5.4 µg/g), dehulled kabog millet sample 2 (320.8 ± 0.3 µg/g), and the reference millet (298.0 ± 2.0 µg/g). Overall, the whole kabog millet samples had significantly higher total phenolic acid content than the dehulled kabog millet samples, the reference millet, and the rice samples (*p* < 0.05) (Table 2).

**Figure 3:**
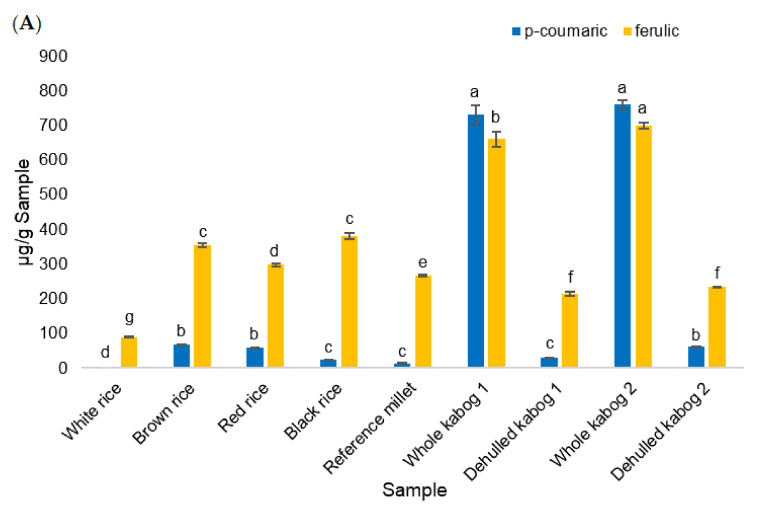

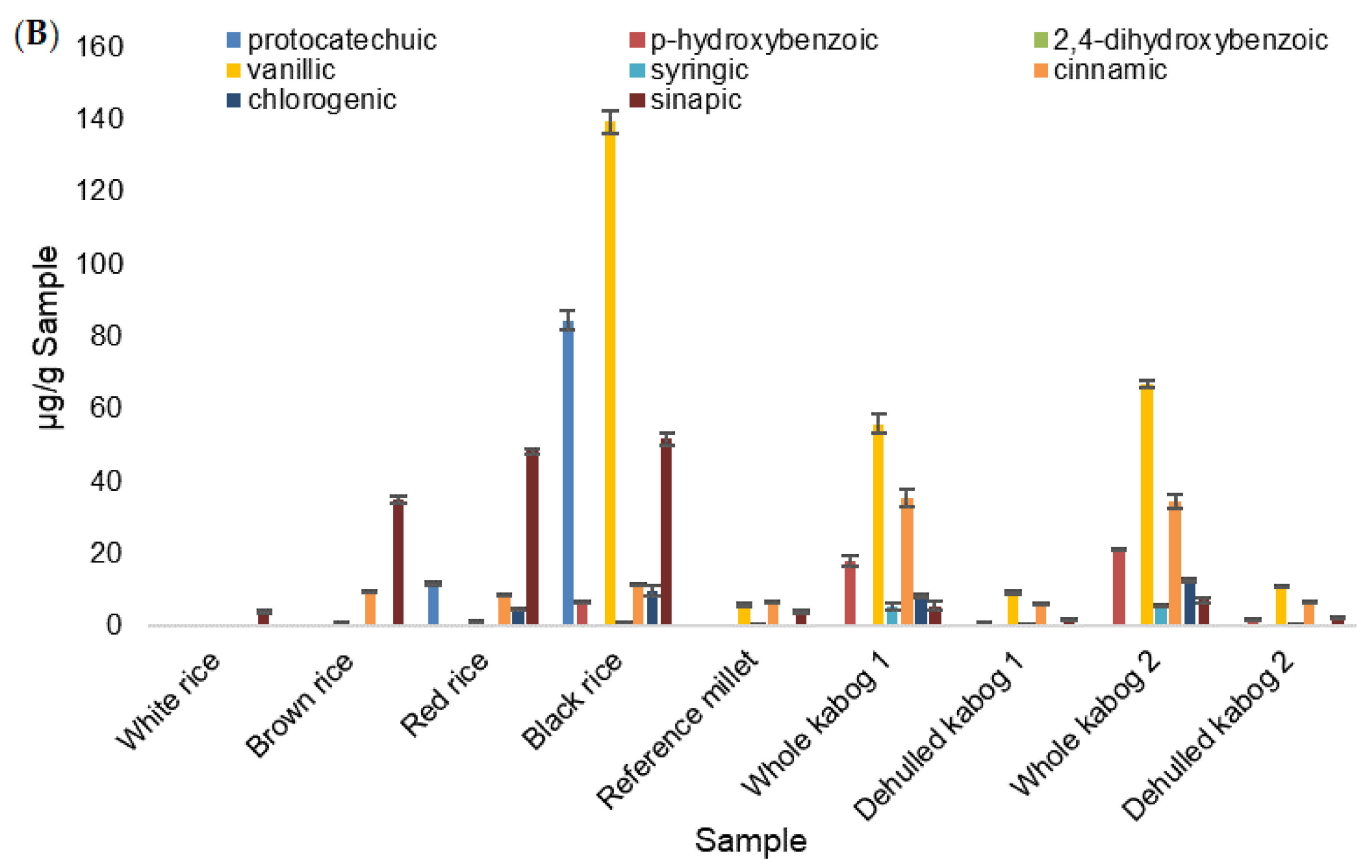
(**A**) Ferulic and *p*-coumaric acid content of rice (white, brown, red, and black), reference millet, and kabog millet (whole and dehulled) samples. (**B**) Individual phenolic acid content (µg/g sample) of rice (white, brown, red, and black), reference millet, and kabog millet (whole and dehulled) samples, not including *p*-coumaric and ferulic acids. Values with the same letters are not significantly different at *p* < 0.05 using one-way ANOVA Tukey’s HSD.

For the individual phenolic acids, the whole kabog millet samples contained the highest amounts of *p-*coumaric and ferulic acids. In whole kabog millet 1, the amounts of *p-*coumaric and ferulic acids were 731 ± 28 and 660 ± 21 µg/g sample, respectively, while the amounts of *p-*coumaric and ferulic acids from sample 2 were 760 ± 13 and 699 ± 9 µg/g sample, respectively. Dehulled kabog millet 1 and 2 contained significantly higher amounts of *p-*coumaric acid (31 ± 1 µg/g and 62 ± 0 µg/g, respectively) than the reference millet (13 ± 1 µg/g) and white rice (undetected) (Figure 3A). The whole kabog millet samples 1 and 2 contained significantly higher amounts of vanillic acid (56 ± 3 µg/g and 67 ± 1 µg/g, respectively) than the reference millet (6 ± 0 µg/g) and white rice (undetected) (*p* < 0.05). The same trend was observed for cinnamic acid, with the whole kabog millet samples 1 and 2 having (35 ± 2 µg/g and 34 ± 2 µg/g, respectively) significantly higher amounts than the reference millet (6 ± 0 µg/g) and white rice (undetected) (*p* < 0.05). On the other hand, black rice had the highest amount of protocatechuic and vanillic acids (84 ± 3 and 139 ± 3 µg/g sample, respectively). White rice contained 90 ± 2 µg/g ferulic acid and 4 ± 0 µg/g sinapic acid (Figure 3B).

### 3.7. Antioxidant Activity

**Figure 4:**
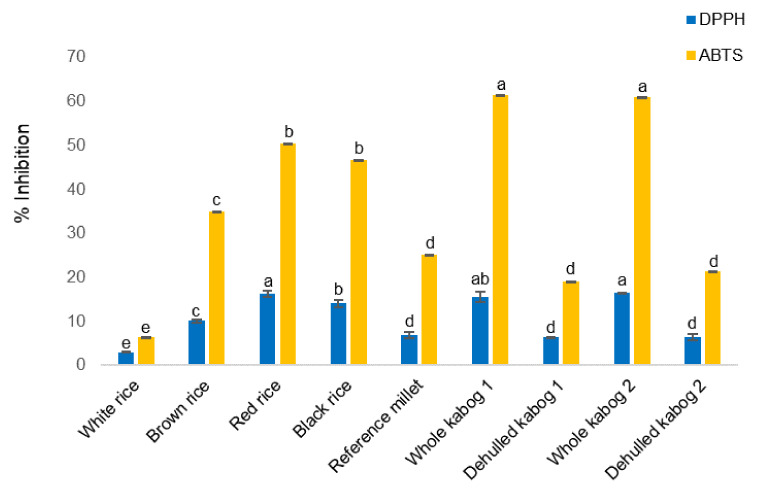
%Inhibition of DPPH (radical scavenging activity, RSA) and ABTS (radical cation scavenging activity) of rice (white, brown, red, and black), reference millet, and kabog millet (whole and dehulled) samples. Values followed by the same letters are not significantly different at *p* < 0.05 using one-way ANOVA Tukey’s HSD.

The total phenolic acid extracts (see Section 2.3.6) were tested for antioxidant activity using DPPH, ABTS, and ORAC assays. In the DPPH assay, the %RSA value of whole kabog millet sample 1 (15.5 ± 1.2%) was not significantly different to that of whole kabog millet 2 (16.4 ± 0.0%) and red rice (16.2 ± 0.6%). However, the dehulled kabog millet samples 1 and 2 had comparable %RSA values (6.3 ± 0.1% and 6.4 ± 0.7%, respectively) as the reference millet (6.9 ± 0.7%) (*p* < 0.05). White rice had the lowest %RSA value (3.0 ± 0.2 %) (Figure 4).

In the ABTS assay shown in Figure 4, the %inhibition values of the ABTS radical cation were not significantly different between the whole kabog millet samples 1 and 2 (61.3 ± 2.6% and 61.3 ± 3.4%, respectively). The %inhibition values of dehulled kabog millet samples 1 and 2 (19.0 ± 0.8% and 22.0 ± 1.7%, respectively) were comparable with the reference millet (25.1 ± 0.7%). The %inhibition values of the whole kabog millet samples were significantly higher than those of red rice (50.4 ± 3.4%), black rice (46.5 ± 3.3%), and brown rice (34.9 ± 2.1%) (*p* < 0.05). The reference millet and the dehulled kabog millet samples had significantly lower %inhibition than red, black, and brown rice samples (*p* < 0.05). The values from the rest of the samples were significantly higher than that of white rice.

In terms of CEAC values, from the ABTS antioxidant assay as shown in Table 3, the whole kabog millet samples 1 and 2 had similar CEAC values (1409.9 ± 60.2 and 1410.7 ± 78.8, respectively). These values were significantly higher than those of red rice (1158.2 ± 79.4), black rice (1068.8 ± 75.8), and brown rice (799.7 ± 49.4) (*p* < 0.05). Meanwhile, the reference millet and the dehulled kabog millet samples 1 and 2 had comparable CEAC values (574.2 ± 16.6, 433.4 ± 19.3, 502.4 ± 40.3, respectively) (*p* < 0.05). These values were significantly lower than those of red, black, and brown rice. With regard to the TEAC values from the ORAC antioxidant assay, the whole kabog millet samples 1 and 2 had similar values (1456.4 ± 14.0 and 1432.3 ± 56.8), which were comparable to those of black rice (1439.2 ± 61.9) and red rice (1312.9 ± 35.3) (*p* < 0.05). Brown rice (1104.2 ± 52.4), the reference millet (1217.9 ± 44.0), and the dehulled kabog millet samples 1 and 2 (1234.2 ± 70.9 and 1160.5 ± 2.9, respectively) had significantly the same TEAC values (*p* < 0.05). White rice had the lowest CEAC and TEAC among all the samples analysed.

### 3.8. Fatty Acid Methyl Esters (FAME)

The fatty acid methyl esters methyl palmitate, methyl stearate, methyl oleate, and methyl linoleate were detected from the whole kabog millet samples 1 and 2. Methyl tricosanoate was also present in small amounts (Supplementary Materials Figure S1). Among the four methyl esters, methyl linoleate had the highest amount in terms of peak area, followed by methyl oleate. Methyl linolenate was not detected by GC-MS.

## 4. Discussion

The total starch values obtained from both whole and dehulled kabog millet samples in our study are comparable with published data for millets. In millets, starch comprises 56% to 65% of the total seed weight, although in proso millet, starch content can be as high as 80% of the total seed weight [16]. This makes the values obtained for the total starch content of the dehulled kabog millet samples within the range for millets. The total starch content values of the whole kabog millet are lower than the rice samples and dehulled kabog millet samples. The relatively low total starch content of the dehulled kabog millet compared to rice can be explained by the rudimentary dehulling process, which removes the hulls that contain starch. Dehulling could also lead to starch damage [17]. The total starch content in rice (white, brown, red, and black) is a good source of energy for people where rice is the major component of their diet [18]. Brown rice typically contains 73% to 76% total starch while white rice has 77% to 78% [19]. A white rice variety, IR64, can have total starch content as high as 84% [20]. In a study of pigmented Thai rice [21], red rice (Red Hommali) has around 81% total starch and black rice (Hom Nin) has around 76% total starch.

The % amylose is important in predicting starch physicochemical properties, such as pasting, gelatinization, and retrogradation properties, and even digestibility [22]. The % amylose values of the kabog millet samples are within the range of those previously reported for three diverse varieties of proso millet [23].The % amylose of the rice types in this study placed them under the low-amylose classification (12 to 20% amylose) [24]. The high amylose content of the reference millet placed it under the non-waxy type millet (≈19% to ≈39% amylose) [25]. Rice with high amylose (above 20%) becomes hard and dry when cooked; hence, lower amylose rice is usually preferred [26]. The difference between the textural properties of the reference millet and the kabog millet upon cooking was not tested in this study, but it can be inferred that similar properties in the reference millet and the kabog millet are possible.

In terms of total dietary fibre content, the whole kabog millet samples have the highest amounts, around three times more than those of brown, red, and black rice, and that of the reference millet, while the dehulled kabog millet samples have comparable values to that of the reference millet (Table 1). This can be attributed to the presence of the hull and the outer bran layer in the whole kabog millet. The hull and the outer bran layer are where the dietary fibre is highly concentrated [19,27]. Likewise, brown rice, which contains the outer bran layer, has higher dietary fibre than white rice [19]. Milling significantly decreases, among other nutrients, the crude fibre and the dietary fibre content [5]. The total dietary fibre contents of white rice and black rice obtained in the current study are within the values reported [28], whilst those of brown rice and red rice from the current study are significantly higher [28]. The difference can be explained by some factors such as rice varieties and sources.

In the present study, the total protein content of the reference millet, the whole kabog millet, and the dehulled kabog millet (≈11% to ≈13%) indicates that they can be good sources of proteins compared to rice and its different types. Rice is not a good source of protein, having only around 7% for white rice and brown rice [28], with brown rice having slightly higher protein content than white rice because of its outer bran layer [19]. Black rice and red rice, which have around 8% protein based on our study, are slightly better alternatives to white rice. Among cereals and pseudocereals, quinoa and amaranth have one of the highest protein contents (≈14% to ≈17%) [15,29]. Millet has around 11% protein [30]. In terms of essential amino acid profiles, quinoa was used as the “gold standard” for the rice and millet samples, because of its good protein quality [15]. In our study, the amounts of the essential amino acids Met, Phe, and Thr in g/100 g protein are comparable in the whole kabog millet and rice, while Lys and Val are higher in rice than in the whole and dehulled kabog millet. On the other hand, Leu is higher in whole and dehulled kabog millet than in rice. Among the essential amino acids, Lys, Trp, and Met are most limiting in cereals [31]. Considering also the amount of total protein, the millet samples have higher essential amino acid contents (g/100 g sample) than the rice samples. Enriching the diet by consuming crop plants (such as cereals and legumes) rich in essential amino acids has both economical and humanitarian interest in developing countries, which still depend on plant-based diets. Kabog millet could play a role in providing essential amino acids to communities that widely consume it.

Carotenoids cannot be synthesised by animals, and hence, are normally obtained through the diet. In terms of carotenoid content, both whole and dehulled kabog millet have the highest lutein and zeaxanthin amounts. β-carotene is also present in the kabog millet samples but is not comparable to black rice, which has the highest β-carotene. In comparison, carrot, a commonly consumed vegetable, is rich in β-carotene and contains lutein, but lacks β-cryptoxanthin and zeaxanthin, whilst red pepper is rich in β-carotene and contains lutein, β-cryptoxanthin, and zeaxanthin [32]. The results of the present study show that the kabog millet samples, particularly the dehulled kabog millet samples, had significantly higher carotenoid values than the previously reported values for finger, little, foxtail, and proso millets [33].

In a previous study [33], HPLC analysis of tocopherols from small millets indicated a higher proportion of γ−and α-tocopherols, but lower levels of tocotrienols. They also reported that δ and γ were prominent in little and proso millets. The content of δ was high in little and proso millets and γ was high in finger and proso millets [33]. These previous findings agree with the results of this study. It shows that whole kabog millet can be a good source of antioxidants in the form of tocopherols. In the case of rice, a study [34] of different varieties of rice in Malaysia revealed that black rice showed significantly higher contents of α-tocopherol, β-tocopherol and α-tocotrienol than non-pigmented rice and red rice. Red rice had significantly lower contents of γ-tocotrienol and total tocopherols than non-pigmented rice. The predominant form of tocopherol isomer in all the varieties analysed was γ-tocotrienol [34]. Similar results were obtained for γ-tocotrienol from the rice samples analysed in the current study; however, β-tocopherol was not detected in our black rice sample, and the total tocopherol content of our red rice sample was not significantly different from that of brown rice (Table 2).

For phenolic acids, the highest amounts of *p-*coumaric and ferulic acids were observed in whole kabog millet. Ferulic acid is found bound to arabinoxylans and other polysaccharides or proteins within the cell walls of the aleurone layer [35,36], which explains its high content in whole kabog millet. Consequently, bound ferulic acid has low bioaccessibility, which limits it applying its systemic health effects [37]. Similar to ferulic acid, *p-*coumaric acid occurs widely in the cell walls of graminaceous plants. The pericarp fractions in barley, wheat, oat, and corn appear to be the fractions richest in *p*-coumaric acid [38]. In millets, *p-*coumaric acid is found in the outer layers of the grain. Milling (or decortication/dehulling) leads to a loss of up to 80% of the total phenolics present in whole grains [39], which explains the low content of *p-*coumaric and ferulic acids in dehulled kabog millet compared to the whole kabog millet samples. Phenolic acids are one of the major bioactives found in brown, red, and black rice grains [19,40]. In a previous study [40], protocatechuic acid was undetectable in white rice. Meanwhile, ferulic and *p*-coumaric are present in red rice, and protocatechuic and vanillic acids are present in black rice. Protocatechuic, vanillic, syringic and ferulic acids have antioxidant activity in the soluble-conjugated fraction, while protocatechuic and ferulic acid are in the insoluble-bound fraction from rice.

Phenolic acids have known antioxidant activities. In addition to that, pigmented rice varieties (red and purple) rich in phenolic acids and anthocyanins were found to have lower starch digestibility than white rice [41]. The interaction of phenolic compounds with starch molecular and/or enzymes could be one factor affecting starch digestibility [42]. This makes phenolic acid- and/or anthocyanin-rich cereals, such as whole kabog millet, possible good sources of low digestible starch for people at high risk of developing diabetes and obesity.

The antioxidant activities of the total phenolic extracts from rice and the millet samples were analysed through DPPH, ABTS, and ORAC antioxidant assays. The DPPH %RSA values are comparable between the whole kabog millet and the red and black rice samples. This makes the phenolic acids found in the whole kabog millet, red rice, and black rice as effective free-radical scavengers. The ability of antioxidant compounds to reduce the ABTS radical cation to its nonradical form was also measure and their activities were compared to Vitamin C (CEAC). The high ABTS %inhibition of whole kabog millet can be correlated to its high total phenolic acid content. In the case of red rice, which has lower total phenolic acid content than brown and black rice, the relatively high ABTS %inhibition cannot be attributed to phenolic acids alone. In a study analysing the antioxidant activity of phenolic acid extracts of millets, it was found that the contents of phenolics alone may not sufficiently explain the observed antioxidant activity of plant phenolic extracts [43]. Other compounds in the extracts could have exerted additional antioxidant activities. In the ORAC assay, the whole kabog millet and black rice phenolics were the most effective peroxyl radical scavengers. In biological systems, peroxyl radicals are stable oxygen-centered radical species formed through autoxidation of fats. This stability enables them to diffuse to distant cellular locations [44].

Cereal lipids are a good source of unsaturated fatty acids. Unsaturated fatty acids can offer several health benefits such as reducing cardiovascular risk, improve cognitive functions and lower GI [45,46]. In a study profiling the fatty acids present in millets, linoleic (18:2) acid was the predominant fatty acid in all millet types (except finger millet) followed by oleic (18:1), palmitic (16:0), stearic (18:0) and linolenic (18:3) acid. Long chain fatty acids namely arachidic (20:0), gadoleic (20:1), behenic (22:0) and lignoceric (24:0) acids were present in all the millet types [47]. In the current study, linoleic acid (in the form of methyl linoleate) was also the predominant fatty acid in kabog millet, followed by oleic (methyl oleate). Linoleic acid is an essential fatty acid and cannot be synthesised in the human body. It serves as a starting point for the synthesis of longer desaturated fatty acids. Tricosanoic acid (in the form of methyl tricosanoate) was also detected in small amounts in whole kabog millet. Tricosanoic acid is also present in foxtail millet [48]. It is interesting to note that tricosanoic acid was present in the lipid extract of kabog millet, while linolenic acid, a common fatty acid in millet grains, was not identified in the GC-MS. Since there is a dearth of information about kabog millet, succeeding studies are needed to completely characterise the lipids present in kabog millet.

One of the special traits of kabog millet is that it is gluten-free, due to its being grouped in the genus *Panicum*. Cereals that contain gluten include wheat, barley, emmer, spelt, einkorn, khorasan, triticale, and rye. Oats contain avenins, which are also considered under the term “gluten” [49]. Cereals and pseudocereals that are gluten-free include rice, maize, sorghum, teff, finger millet, pearl millet, foxtail millet, buckwheat, amaranth, and quinoa. About 1% to 2% of the general population have adverse inflammatory reactions to gluten, called celiac disease (CD), while non-celiac gluten sensitivity (NCGS) is estimated to be six to ten times higher than celiac disease [50]. A gluten-free diet is the most common therapy for both CD and NCGS.

Some of the food products from gluten-free cereals and pseudocereals that are currently gaining ground are sourdough bread and gluten-free beers. For sourdough bread, African cereals called Acha or white fonio (*Digitaria exiliis*) and Iburu or black fonio (*Digitaria iburua*) have been used and studied [51]. Millet- and sorghum-based fermented foods that rely on lactic acid bacteria (LAB) are also produced daily in Africa [52]. Teff, a gluten-free cereal native to Ethiopia, is an ingredient of a sour fermented flatbread called enjerra (injera) [53]. The advantages of using sourdough in bread-making are due to the fermentative and acidifying activity of LAB and yeasts [54]—such processes include lactic acid fermentation, proteolysis, exopolysaccharides production, and synthesis of compounds with antimicrobial activities. These metabolic activities enhance gluten-free bread properties such as texture, flavor, taste, volume, and nutritional quality [55]. Fermentation also reduces the anti-nutritive factors found in cereals and prolongs food stability [52,56]. Because it is gluten-free, kabog millet can be used to develop food products targeted to CD and NCGS sufferers. No study yet has been performed on the nutritional and sensory characteristics of sourdough bread made using kabog millet flour. This could be an avenue for further investigation.

### Limitations to the Study

There were some limitations in the samples used in this study, namely, the kabog millet, the reference millet, and the rice samples were not cultivated under similar conditions or in the same country. Kabog millet samples were also grown within the same year but in different agro-climatic conditions. The kabog millet used in the study cannot be cultivated outside the Philippines under the existing Material Transfer Agreement (MTA). In addition, except for the kabog millet and the dawa millet, millets are not common cereals in the Philippines. Even then, the cultivation of these two native millets is concentrated in specific areas only, and these millets are not widely known in the Philippines. Another limitation is the use of quinoa data from previous literature [15] to compare the essential amino acids of the rice, the reference millet, and the kabog millet samples.

## 5. Conclusions

Based on the results of this study, and considering the limitations, kabog millet, particularly the whole grain samples, were found to possess good nutritional quality in terms of total dietary fiber and total protein content, essential amino acid profiles, phenolic acid, tocopherol, and carotenoid content, fatty acid profile, and antioxidant activity by DPPH, ABTS, and ORAC assays. It can be promoted as a healthier cereal substitute to white rice in the Philippines and potentially, in other rice-consuming countries in the world. It is hoped that increased consumption of kabog millet will lead to the conservation of its genetic pool for future generations, and the rich cultural heritage of the kabog millet-growing regions in the Philippines. It will also provide livelihood to small-scale farmers and their families. More consumers will appreciate the global diversity of cereal crops and will have access to nutritional and sustainable food choices by incorporating kabog millet into their diet.

## Figures and Tables

**Table 1:** Total starch, % amylose, dietary fibre, ash, and total protein content of rice (white, brown, red, and black), reference millet, and kabog millet (whole and dehulled) samples expressed as percentages.

Samples ^1^	Total Starch	% Amylose	Dietary Fibre	Ash	Protein ^2^ (g/100 g)
White rice	81.5 ± 0.7 ^a^	16.5 ± 1.6 ^b^	1.0 ± 0.4 ^c^	0.3 ± 0.0 ^g^	6.4 ± 0.0 ^h^
Brown rice	76.2 ± 1.0 ^a^	18.4 ± 0.1 ^b^	4.3 ± 0.7 ^b^	1.3 ± 0.0 ^d^	7.2 ± 0.0 ^g^
Red rice	71.8 ± 1.9 ^b^	18.7 ± 1.1 ^b^	5.6 ± 0.7 ^b^	1.3 ± 0.0 ^d^	8.0 ± 0.0 ^f^
Black rice	72.6 ± 2.1 ^b^	16.5 ± 0.6 ^b^	5.0 ± 0.5 ^b^	1.6 ± 0.0 ^c^	8.4 ± 0.0 ^e^
Reference millet	72.3 ± 0.2 ^b^	42.3 ± 1.9 ^a^	4.4 ± 0.7 ^b^	1.1 ± 0.0 ^e^	11.1 ± 0.1 ^d^
Whole kabog 1	51.6 ± 1.8 ^d^	16.4 ± 1.1 ^b^	14.6 ± 0.6 ^a^	4.2 ± 0.0 ^a^	12.0 ± 0.0 ^c^
Dehulled kabog 1	60.1 ± 2.7 ^c^	19.1 ± 1.8 ^b^	3.6 ± 0.6 ^b^	1.0 ± 0.0 ^f^	12.5 ± 0.0 ^b^
Whole kabog 2	47.4 ± 2.0 ^d^	18.1 ± 1.0 ^b^	13.8 ± 0.9 ^a^	4.1 ± 0.1 ^b^	12.1 ± 0.0 ^c^
Dehulled kabog 2	63.2 ± 1.7 ^c^	10.7 ± 1.3 ^c^	4.0 ± 1.1 ^b^	1.1 ± 0.0 ^e^	12.9 ± 0.1 ^a^

^1^ Data is expressed as Mean ± SD (*n* = 3). ^2^ Protein content was analysed by Dumas method. Values followed by the same letters are not significantly different at *p* < 0.05 using Tukey’s HSD. The different superscript letters represent significance at *p* < 0.05 using Tukey’s HSD.

**Table 2:** Total carotenoid, tocopherol, and phenolic acid content of rice (white, brown, red, and black), reference millet, and kabog millet (whole and dehulled) samples expressed in µg/g.

Samples ^1^	Carotenoids	Phenolic Acids	Tocopherols
White rice	0.2 ± 0.0 ^(2) e^	93.9 ± 2.3 ^f^	10.8 ± 0.1 ^d^
Brown rice	0.9 ± 0.0 ^(2) e^	467.5 ± 7.0 ^d^	52.7 ± 1.7 ^c^
Red rice	1.0 ± 0.0 ^(2) e^	433.0 ± 4.8 ^d^	58.2 ± 4.5 ^c^
Black rice	3.5 ± 0.1 ^d^	707.0 ± 13.9 ^c^	120.0 ± 15.7 ^a^
Reference millet	6.9 ± 0.5 ^c^	298.0 ± 2.0 ^e^	37.2 ± 2.0 ^c,d^
Whole kabog 1	16.8 ± 0.4 ^b^	1519.0 ± 47.1 ^b^	93.1 ± 9.0 ^b^
Dehulled kabog 1	19.4 ± 0.3 ^a^	264.7 ± 5.4 ^e^	45.0 ± 5.3 ^c^
Whole kabog 2	15.7 ± 0.6 ^b^	1606.6 ± 16.2 ^a^	99.4 ± 9.4 ^a,b^
Dehulled kabog 2	19.3 ± 0.5 ^a^	320.8 ± 0.3^(2) e^	50.4 ± 6.3 ^c^

**^1^** Data is expressed as Mean ± SD (*n* = 3) except ^(**2**)^ where *n* = 2. Values followed by the same letters are not significantly different at *p* < 0.05 using Tukey’s HSD. The different superscript letters represent significance at *p* < 0.05 using Tukey’s HSD.

**Table 3:** CEAC and TEAC values in the ABTS and ORAC antioxidant assays, respectively, of rice (white, brown, red, and black), reference millet, and kabog millet (whole and dehulled) samples.

Sample ^1^	CEAC (ABTS)	TEAC (ORAC)
White rice	141.9 ± 30.5 ^e^	173.6 ± 2.3 ^d^
Brown rice	799.7 ± 49.4 ^c^	1104.2 ± 52.4 ^c^
Red rice	1158.2 ± 79.4 ^b^	1312.9 ± 35.3 ^a,b^
Black rice	1068.8 ± 75.8 ^b^	1439.2 ± 61.9 ^a^
Reference millet	574.2 ± 16.6 ^d^	1217.9 ± 44.0 ^b,c^
Whole kabog 1	1409.9 ± 60.2 ^a^	1456.4 ± 14.0 ^a^
Dehulled kabog 1	433.4 ± 19.3 ^d^	1234.2 ± 70.9 ^b,c^
Whole kabog 2	1410.7 ± 78.8 ^a^	1432.3 ± 56.8 ^a^
Dehulled kabog 2	502.4 ± 40.3 ^d^	1160.5 ± 2.9 ^b,c^

^1^ Data is expressed as Mean ± SD (*n* = 3). Values followed by the same letters are not significantly different at *p* < 0.05 using Tukey’s HSD. The different superscript letters represent significance at *p* < 0.05 using Tukey’s HSD.

## Supplementary Materials

The following are available online at https://www.mdpi.com/2304-8158/9/12/1727/s1, Figure S1: Fatty acid methyl esters (FAMEs) analysis by GC-MS showing the total ion chromatogram (TIC) of fatty acids extracted from whole kabog millet sample source 1 (A) and whole kabog millet sample source 2 (B). (*****) in methyl tricosanoate refers to very small quantities detected.
