# Supplementary material for: Breathing New Life to Ancient Crops: Promoting the Ancient Philippine Grain “Kabog Millet” as an Alternative to Rice
Source: Foods. 2020 Nov 24;9(12):1727. doi: 10.3390/foods9121727 (PMC7761226; doi:10.3390/foods9121727)
Supplement: Supplementary file 1 [file foods-09-01727-s001.pdf]

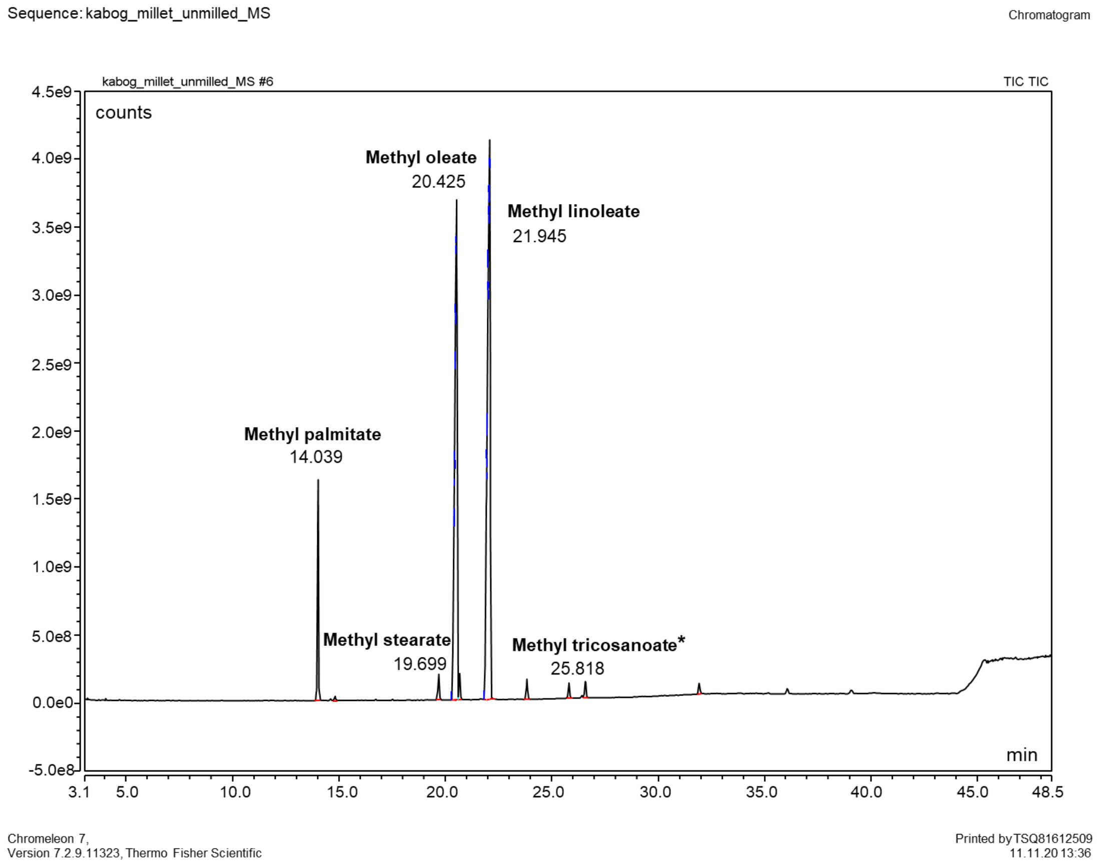

(**A**)

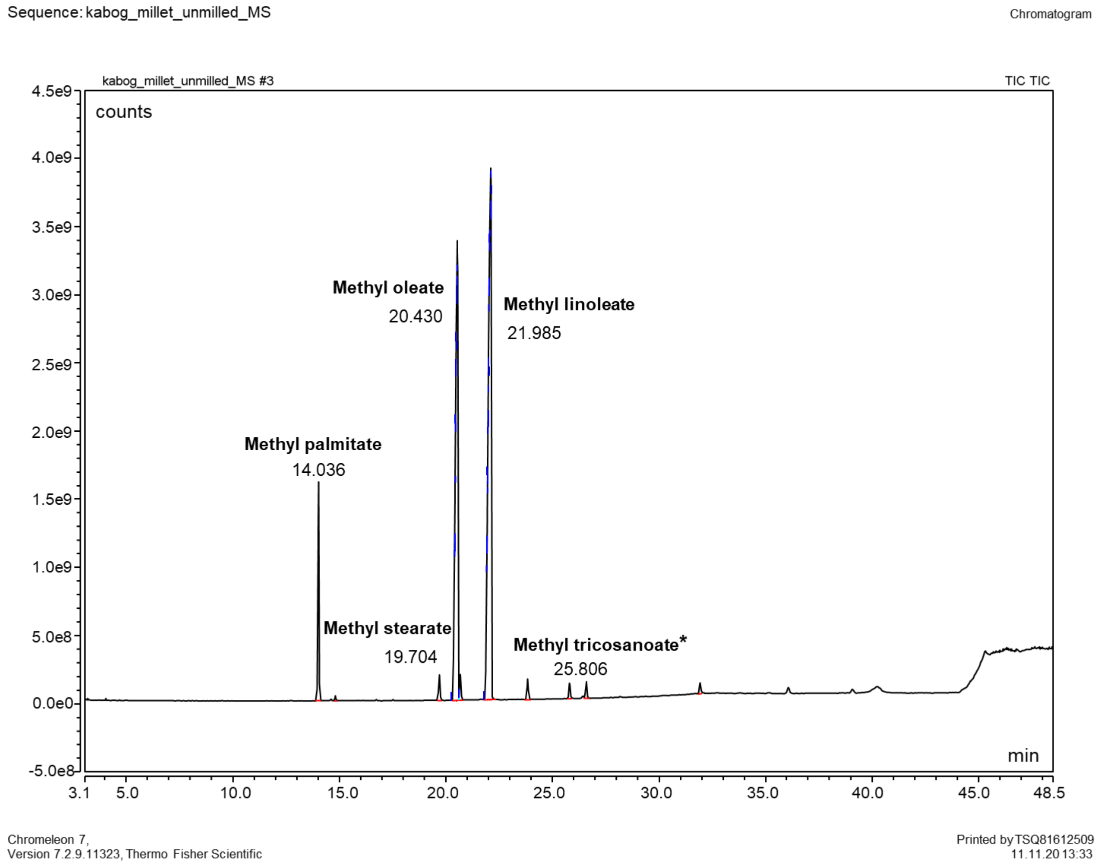

(**B**)

Figure S1. Fatty acid methyl esters (FAMEs) analysis by GC-MS showing the total ion chromatogram (TIC) of fatty acids extracted from whole kabog millet sample source 1 (**A**) and whole kabog millet sample source 2 (**B**). (*) in methyl tricosanoate refers to very small quantities detected.
